# Effects of Exercise on Parkinson’s Disease: A Meta-Analysis of Brain Imaging Studies

**DOI:** 10.3389/fnhum.2022.796712

**Published:** 2022-02-16

**Authors:** Jingwen Li, Jian Guo, Weijuan Sun, Jinjin Mei, Yiying Wang, Lihong Zhang, Jianyun Zhang, Jing Gao, Kaiqi Su, Zhuan Lv, Xiaodong Feng, Ruiqing Li

**Affiliations:** ^1^College of Rehabilitation Medicine, Henan University of Chinese Medicine, Zhengzhou, China; ^2^Rehabilitation Center, The First Affiliated Hospital of Henan University of Chinese Medicine, Zhengzhou, China

**Keywords:** exercise, Parkinson’s disease, brain imaging, meta-analysis, activation likelihood estimation

## Abstract

**Background:**

Exercise is increasingly recognized as a key component of Parkinson’s disease (PD) treatment strategies, but the underlying mechanism of how exercise affects PD is not yet fully understood.

**Objective:**

The activation likelihood estimation (ALE) method is used to study the mechanism of exercise affecting PD, providing a theoretical basis for studying exercise and PD, and promoting the health of patients with PD.

**Methods:**

Relevant keywords were searched on the PubMed, Cochrane Library, and Web of Science databases. Seven articles were finally included according to the screening criteria, with a total sample size of 97 individuals. Using the GingerALE 3.0.2 software, an ALE meta-analysis was performed using seven studies that met the requirements, and the probability of the cross-experiment activation of each voxel was calculated.

**Results:**

The meta-analysis produced seven clusters, and major activations were found in the cerebellum, occipital lobe, parietal lobe, and frontal lobe brain regions.

**Conclusion:**

Exercise for PD mainly results in the enhanced activation of the cerebellum, occipital lobe, parietal lobe, and frontal lobe. Exercise for PD does not cause a change in the activation of a single brain area, and the observed improvement may result from coordinated changes in multiple brain areas.

## Introduction

Parkinson’s disease (PD) is the second most common progressive neurodegenerative disorder, affecting 2–3% of older adults. Its incidence and prevalence are highest among people aged ≥65 years, making the disease a significant public health burden for older adults (Poewe et al., 2017). The clinical symptoms of PD include tremors, rigidity, bradykinesia, and postural instability. In addition to these motor impairments, patients may also experience various non-motor symptoms, including psychiatric symptoms. Both of these symptom types increase mortality, profoundly affecting human health and quality of life (Takamiya et al., 2021).

The main treatments for PD involve pharmacological and surgical interventions; however, some patients are not sensitive or responsive to levodopa drugs, and some may develop motor complications after the long-term use of dopaminergic drugs. The symptoms of PD are difficult to adequately control with these treatments due to the incurable and progressively neurodegenerative nature of diseases (Álvarez-Bueno et al., 2021; Ge et al., 2021). Currently, exercise is increasingly recognized as a key component in the treatment strategy for PD and has received significant attention due to its easy accessibility, low cost, and low technical equipment requirements (Ji et al., 2021; Morley et al., 2021). Many clinical studies and meta-analyses have shown that exercise promotes the recovery of motor and non-motor symptoms in patients with PD (da Silva et al., 2018). For example, implementing an exercise program improves the gait, balance, and motor capacity of patients with PD and their quality of life (Gilat et al., 2021), and resistance exercises can promote neuroplastic changes within the central nervous system of patients with PD to improve cognitive functioning (Chow et al., 2021).

The mechanism of exercise for treating PD may be related to changes in the dopaminergic system, brain, and glial-derived neurotrophic factors, and the modulation of neuroinflammation (Earhart and Falvo, 2013). In addition, it may also increase neurotrophic signaling and promote neurogenesis by changing the cerebral vascular system (Sacheli et al., 2019). Although the therapeutic mechanism of exercise has been broadly described, most studies only discuss this at the molecular level, and the neurobiological mechanism remains unclear. In recent years, with the continuous progress in functional imaging technologies, an increasing number of studies have explored the mechanism of exercise for treating PD through neuroimaging technology (Weingarten et al., 2015; Myers et al., 2018). Among them, functional MRI (fMRI) is widely used due to its advantages of being non-invasive, non-radioactive, and having a relatively high spatial resolution, which allows the study of PD to be extended to the level of brain function/neurotic material metabolism and to understand further the pathogenesis of PD motor and non-motor symptoms (Bidesi et al., 2021; Mitchell et al., 2021).

The activation likelihood estimation (ALE) is one of the most commonly used meta-analysis methods in the field of brain imaging in recent years (Eickhoff et al., 2010). The basic principle of ALE is to calculate the probability that each voxel is activated under certain conditions in each experiment and to statistically analyze these data (Wager et al., 2007). Using the activation probability as an indicator, the probability of the cross-experiment activation of each voxel was calculated, and the hypothesis testing was performed on this possibility to obtain a general conclusion of the execution control of the relevant brain activation area in multiple experiments (Isherwood et al., 2021).

The purpose of our study was to review the published literature on exercise intervention for PD and to use ALE for analyzing the included literature, exploring the neural mechanism of exercise affecting PD, expanding our knowledge of exercise and PD-related brain science research, and deepening our understanding of neuroscience. We aimed to provide a theoretical basis for exercise in promoting the health of patients with PD.

## Materials and Methods

### Literature Search

This meta-analysis was conducted according to the Preferred Reporting Items for Systematic Reviews and Meta-Analyses (PRISMA) guidelines (Shamseer et al., 2015).

We searched the PubMed, Cochrane Library, and Web of Science databases for relevant articles published on September 20, 2021, using exercise-related keywords such as “exercise, training, physical activity, physical therapy, fitness, traditional Chinese exercise, swimming, yoga, running, walking, cycling, and Tai Chi,” PD-related keywords such as “Parkinson and PD,” and neuroimaging-related keywords such as “fMRI, functional magnetic resonance imaging, neuroimaging, brain, cortical, and neural.” There were no restrictions on language or publication status. The reference lists of studies were also reviewed to identify other relevant eligible studies.

### Inclusion and Exclusion Criteria

The inclusion criteria for this study were in the order of the population, intervention, comparator, outcome, and study design (PICOS) formulation.

1) Studies clearly describing patients suffering from PD (diagnosed using established clinical diagnostic criteria);
2) The intervention involved any form of exercise during the hospital course of PD. Examples of well-recognized types of exercise include training, physical activity, physical therapy, fitness, traditional Chinese exercise, swimming, yoga, running, walking, cycling, and Tai Chi;
3) The comparators were those who did not undergo exercise intervention during PD;
4) Studies which included functional neuroimaging measurement;
5) Only literature that used standardized Montreal Neurological Institute (MNI) or Talairach coordinates to represent the peak coordinates of the brain area;
6) Studies using whole-brain analysis instead of a region of interest (ROI) analysis;
7) Observational studies, randomized controlled trials, and clinical trials were included.

The exclusion criteria were as follows: literature reviews, animal studies, studies involving *in vitro* experiments, and non-randomized controlled studies.

Two researchers independently conducted a systematic review using the same criteria and included the study based on agreement. When there was a disagreement, a third investigator joined and helped make the final decision.

### Data Extraction

Data extraction was performed independently from the included studies using an established data collection form. The following information was obtained: name of the author, publication year, patient information (diagnostic criteria and age), treatment, imaging conditions, and coordinates provided in standard space (i.e., Talairach and Tournoux or MNI space).

### Statistical Analysis

All included studies were assessed using the Covidence Quality Assessment Template after a quality check. In this study, GingerALE 3.0.2 and Mango 4.1 software were used to analyze the data.

Ginger ALE 3.0.2 software was used for ALE meta-analysis. The ALE meta-analysis converts the coordinates reported in the MNI space standard to Talairach coordinates using Lancaster, which is carried out under the Talairach space standard. At the same time, the inference was made at the cluster level, resulting in a better balance between the sensitivity and specificity of the study (Eickhoff et al., 2012). According to the activation coordinates, a three-dimensional Gaussian model was used to establish an ALE map. Based on the recommendations of the ALE instruction manual, the cluster-forming threshold was set at *P* < 0.05, to overcome the false-positive problems of multiple comparisons, the false discovery rate method was used for correction, and the minimum cluster volume was set to 200 mm^3^. Using Mango 4.1 software, the threshold ALE image was superimposed on the standardized anatomical template in the Talairach space to visualize the results.

In the case of a small number of included studies, implementing a meta-analysis requires a certain degree of vigilance (Valentine et al., 2010). To avoid bias, we distinguished significant results from insignificant peaks, and only clustering results are discussed in detail in our study.

## Results

### Study Selection

**FIGURE 1:**
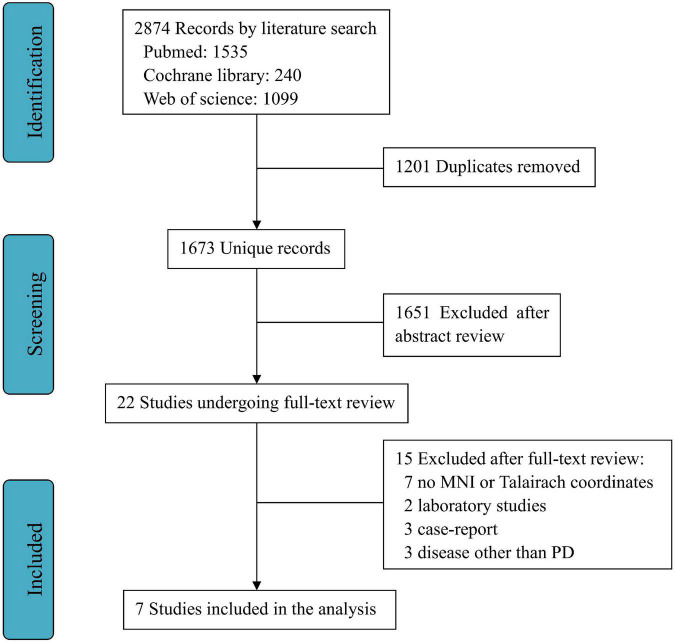
Workflow for selection process.

A total of 2,874 publications were selected through databases for initial screening, and among them, 1,201 duplicates were removed, and 1,651 articles were excluded as they did not satisfy the inclusion criteria. As a result, through the full text of 22 screened studies, only seven fully satisfied all the criteria for inclusion in the meta-analysis (Shine et al., 2013; Duchesne et al., 2016; Agosta et al., 2017; Kelly et al., 2017; Messa et al., 2019; Segura et al., 2020; Silva-Batista et al., 2020). A flowchart of this process is shown in Figure 1.

### Main Clusters

**FIGURE 2:**
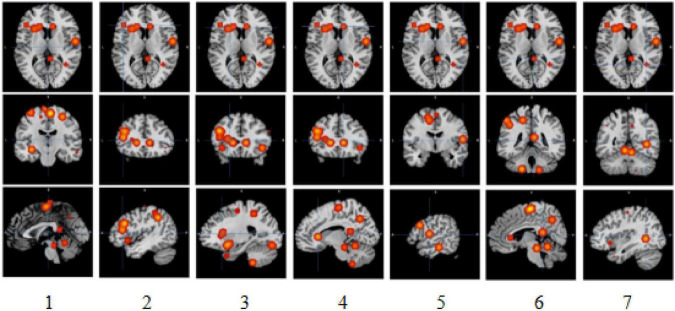
Slice view of clusters. 1, 2, 3, 4, 5, 6, and 7, respectively, indicate activation clusters that have reached a significant activation level.

The meta-analysis yielded six clusters with four peaks in the first and second clusters and two peaks in the other clusters. The full results are detailed in Tables 1, 2 and Figure 2.

**TABLE 1:** Characteristics of the 7 included studies.

Study	Number of participants	Mean age (SD)	Treatment	Imaging conditions	Coordinates
Segura et al., 2020	6	57.8	Bicycle	fMRI	MNI
Duchesne et al., 2016	19	59 (7.11)	Exercise	fMRI	MNI
Silva-Batista et al., 2020	15	64.6 (10.5)	Resistance training	fMRI	MNI
Kelly et al., 2017	17	66.6 (5.8)	Exercise	fMRI	MNI
Agosta et al., 2017	12	69.0 (8.0)	Exercise	fMRI	MNI
Messa et al., 2019	10	69.1 (6.5)	Exercise	fMRI	MNI
Shine et al., 2013	18	66.8 (8.2)	Walking	fMRI	MNI

**TABLE 2:** Clusters and peaks.

Cluster #	x	y	z	ALE	Area	Brodmann	Hemisphere
1	−44	24	28	0.009119155	Frontal lobe. Middle frontal gyrus.	9	L
1	−16	24	4	0.00773276	Sub-lobar. Caudate.		L
1	−44	28	14	0.007248954	Frontal lobe. Middle frontal gyrus.	46	L
1	−26	20	10	0.004873995	Sub-lobar. Claustrum.		L
2	4	−16	62	0.010242551	Frontal lobe. Medial frontal gyrus.	6	R
2	−6	−22	72	0.007914808	Frontal lobe. Medial frontal gyrus.	6	L
2	−12	−4	50	0.005071956	Frontal lobe. Medial frontal gyrus.	6	L
2	−14	−10	56	0.00495608	Frontal lobe. Medial frontal gyrus.	6	L
3	−18	6	−12	0.008865426	Sub-lobar. Lentiform nucleus.		L
3	−38	16	−8	0.007668495	Sub-lobar. Insula.	13	L
4	−6	−52	−12	0.007571907	Anterior lobe. Culmen.		L
4	6	−52	−16	0.007520604	Anterior lobe. Culmen.		R
5	24	−85	−10	0.0049557	Occipital lobe. Fusiform gyrus.	19	R
5	27	−82	−1	0.004842124	Occipital lobe. lingual gyrus.	18	R
6	−46	−46	40	0.00796068	Parietal lobe. Supramarginal gyrus.	40	L
6	−52	−40	50	0.007166639	Parietal lobe. Inferior parietal lobule.	40	L
7	−21	−82	−16	0.0049557	Posterior lobe. declive.		L
8	60	−26	−18	0.007731717	Temporal lobe. Middle temporal gyrus.	21	R
9	58	−6	10	0.007144281	Frontal lobe. Precentral gyrus.	43	R
10	6	−42	14	0.006627638	Limbic lobe. Posterior cingulate.	29	R
11	8	−62	40	0.006627638	Parietal lobe. Precuneus.	7	R
12	−18	−44	50	0.006627638	Parietal lobe. Precuneus.	7	L
13	30	−16	56	0.007144281	Frontal lobe. Precentral gyrus.	4	R
14	−33	−67	−19	0.004731151	Posterior lobe. Declive.		L
15	11	25	3	0.007424954	Sub-lobar. Caudate.		R
16	6	−30	−19	0.007497888			
17	45	20	−8	0.007913446	Sub-lobar. Insula.	13	R
18	38	−54	1	0.007787312	Limbic lobe. Parahippocampal gyrus.	19	R
19	−36	−19	65	0.004842124	Frontal lobe. Precentral gyrus.	4	L
20	−30	12	−38	0.008291761	Temporal lobe. Superior temporal gyrus.	38	L
21	16	−46	−54	0.008291758			
22	−20	−42	−52	0.008291758	Posterior lobe. Cerebellar tonsil.		L
23	28	−72	−38	0.008291758	Posterior lobe. Inferior semi-lunar lobule.		R
24	30	12	−36	0.008291758	Temporal lobe. Superior temporal gyrus.	38	R
25	−34	−16	−12	0.008291758	Temporal lobe. Caudate.		L
26	60	14	28	0.008169615	Frontal lobe. Inferior frontal gyrus.	9	R

The first cluster from (*x*, *y*, *z* = −52, 12, −4) to (*x*, *y*, *z* = −8, 36, 36), centered at (*x*, *y*, *z* = −34.5, 25.1, 16.6) with four peaks and a max value at (*x*, *y*, *z* = −44, 24, 28) (ALE = 0.0091, *P* = 4.2159907 × 10^–5^, *Z* = 3.93), was located in the left brain hemisphere, with 56.9% frontal lobe activation and 43.1% sub-lobar activation. Anatomically, this corresponds to the middle frontal gyrus (40.5%), caudate (31.8%), precentral gyrus (10.6%), claustrum (7.7%), inferior frontal gyrus (5.8%), and insula (3.3%). In terms of functional areas, these were mainly Brodmann area 9 (44.9%), the caudate body (21.5%), the caudate head (10.2%), Brodmann area 46 (9.1%), Brodmann area 13 (3.3%), and Brodmann area 45 (2.9%). This corresponds to the activation of the left cerebrum frontal lobe in the middle frontal gyrus.

The second cluster from (*x*, *y*, *z* = −22, −30, 42) to (*x*, *y*, *z* = 14, 4, 78), centered at (*x*, *y*, *z* = −3.6, −13.8, 60.7) with four peaks and a max value at (*x*, *y*, *z* = 4, −16, 62) (ALE = 0.0102, *P* = 1.7117862 × 10^–5^, *Z* = 4.14), was located in the left brain hemisphere (60.1%) and right brain hemisphere (39.9%), with 85.8% frontal lobe activation, 13% limbic lobe activation, and 1.2% parietal lobe activation. Anatomically, this corresponds to the medial frontal gyrus (70%), paracentral lobule (14.4%), cingulate gyrus (12.3%), postcentral gyrus (1.4%), and precentral gyrus (1.4%). In terms of functional areas, these are mainly Brodmann area 6 (81.5%), Brodmann area 24 (10.3%), Brodmann area 31 (5.3%), Brodmann area 3 (1.4%), and Brodmann area 4 (1.4%). This corresponds to activation of the left and right cerebrum, frontal lobe, and medial frontal gyrus.

The third cluster from (*x*, *y*, *z* = −46, −2, −24) to (*x*, *y*, *z* = −10, 24, 0), centered at (*x*, *y*, *z* = −26.8, 11.6, −12) with two peaks and a max value at (*x*, *y*, *z* = −18, 6, −12) (ALE = 0.0089, *P* = 5.1213978 × 10^–5^, *Z* = 3.88), was located in the left brain hemisphere, with 77.5% sub-lobar activation and 21.8% frontal lobe activation. Anatomically, this corresponds to the lentiform nucleus (53.5%), inferior frontal gyrus (16.9%), insula (16.4%), subcallosal gyrus (5.2%), extranuclear (3.1%), and claustrum (3.1%). In terms of functional areas, these were mainly putamen (42%), Brodmann area 47 (18.9%), Brodmann area 13 (16.6%), globus pallidus (10.8%), and Brodmann area 34 (3.8%). This corresponds to the activation of the left cerebrum sub-lobar lentiform nucleus.

The fourth cluster from (*x*, *y*, *z* = −14, −60, −24) to (*x*, *y*, *z* = 14, −44, −4), centered at (*x*, *y*, *z* = −0.1, −52.5, −14) with two peaks and a max value at (*x*, *y*, *z* = −6, −52, −12) (ALE = 0.0076, *P* = 2.712689 × 10^–4^, *Z* = 3.46), was located in the left cerebellum (58.2%) and right cerebellum (41.8%), with 97% anterior lobe activation and 3% posterior lobe activation. Anatomically, this corresponds to the culmen (76.8%), cerebellar lingual (18.9%), declive (3%), and fastigium (1.3%). This corresponds to activations of the left and right cerebellum anterior lobe culmen.

The fifth cluster from (*x*, *y*, *z* = 16, −92, −18) to (*x*, *y*, *z* = 34, −74, 6), centered at (*x*, *y*, *z* = 25.5, −83.5, −5.5) with two peaks and a max value at (*x*, *y*, *z* = 24, −85, −10) (ALE = 0.005, *P* = 0.0041320226, *Z* = 2.64), was located in the right brain hemisphere (62%) and right cerebellum (38%), with 62% occipital lobe activation and 38% posterior lobe activation. Anatomically, this corresponds to the lingual gyrus (38.9%), live (38%), fusiform gyrus (15.8%), middle occipital gyrus (5.4%), and inferior occipital gyrus (1.4%). In terms of functional areas, these were mainly Brodmann area 18 (46.6%) and Brodmann area 19 (15.4%). This corresponds to the activation of the right cerebrum and right cerebellum occipital lobe, lingual gyrus, and posterior lobe lingual declive.

The sixth cluster from (*x*, *y*, *z* = −58, −54, 32) to (*x*, *y*, *z* = −38, −32, 56), centered at (*x*, *y*, *z* = −48, −43.2, 44.4) with two peaks and a max value at (*x*, *y*, *z* = −46, −46, 40) (ALE = 0.008, *P* = 1.3964054 × 10^–4^, *Z* = 3.63), was located in the left brain hemisphere, with parietal lobe activation. Anatomically, this corresponds to the inferior parietal lobule (82.2%) and supramarginal gyrus (17.8%). The functional area involved here was Brodmann area 40 (100%). This corresponds to the activation of the left cerebrum, parietal lobe, and inferior parietal lobule.

## Discussion

Our study used an ALE meta-analysis to elucidate that the activation of brain regions affected by exercise in PD is mainly located in the left and right cerebellum, left middle frontal gyrus, left and right medial frontal lobes, right occipital lobes, and left and right parietal lobes.

### Effect of Exercise on the Cerebellum of Patients With Parkinson’s Disease

The cerebellum is the main structure that affects movement, cognition, and emotional behavior. It is well known that the cerebellum affects motor and cognitive functions through the cerebellar-thalamic-cortical circuit (Pierce and Péron, 2020). Studies have shown that multiple areas of the cerebellum in patients with PD are atrophied, the subthalamic nucleus has lost its ability to regulate connections in the cerebellum, and there are extensive cerebellar-cortical network abnormalities (Shi et al., 2021). In patients with PD, the volume of cerebellar gray matter decreases, the connections within the cerebellum and between the cerebellum and the sensory-motor network increase, the connections between the cerebellum and the caudate nucleus, thalamus, and amygdala increase, and the connections between the auxiliary motor area and the cingulate gyrus decrease (Tuovinen et al., 2018). In rats with PD following aerobic exercise, the functional reorganization of brain activity was observed in the cerebellum, thereby improving their exercise capacity (Wang et al., 2015). Neuroimaging studies have also found that patients with PD change the activation of the cerebellum during exercise, motor learning, and rest. The cerebellum and its circuits play a vital role in PD tremors (Duchesne et al., 2016). Therefore, exercise may reorganize the function of the cerebellum and its circuits to improve motor function and cognitive dysfunction after PD.

### Effect of Exercise on the Occipital, Frontal, and Parietal Lobes of the Brain in Patients With Parkinson’s Disease

The occipital lobe is highly related to basic cognitive processing, such as visual deficits and visual attention (Göttlich et al., 2013). Studies have found that the executive function is mainly related to the metabolism of the parieto-occipital junction and frontal lobe, the mnemonic function is related to the metabolism of the parietal lobe, the visuospatial function is related to the occipital parietal lobe, and language is related to the metabolism of the frontal lobe. Decreased metabolism in the frontal and parietal regions and the parieto-occipital lobe can lead to executive dysfunction and cognitive impairment in patients with PD (Abe et al., 2003; Garcia-Garcia et al., 2012). Learning motor tasks in patients with PD mainly relies on the compensatory cortical circuit involving the prefrontal lobe area, and it also produces changes in the brain activation of the cerebellum and prefrontal lobe area. Physical exercise affects the plasticity of the brain and has a significant impact on the parietal and frontal cortex, as well as the anterior and transverse tracts between the frontal and parietal regions, i.e., the areas involved in cognition and daily life functions (Maidan et al., 2017; da Silva et al., 2018). Therefore, exercise may affect the frontal cortex circuit and parieto-occipital frontal metabolism, thereby affecting brain plasticity and improving the non-motor symptoms of PD.

From the abovementioned analysis, it can be observed that the cerebellum, occipital lobe, parietal lobe, and frontal lobe brain areas are the important areas for exercise interventions in the treatment of PD. There is no current consensus on the neural mechanism of exercise that promotes the recovery of PD. This study performed an ALE meta-analysis of the included literature and achieved cross-experimental consistency, which not only overcomes the limitations of a single study but also increases statistical power, and clarifies the neural mechanism of exercise to improve PD; i.e., exercise that enhances the activation of the cerebellum, occipital lobe, parietal lobe, and frontal lobe promotes the improvement of PD.

This meta-analysis included seven studies on the brain activation patterns of PD that met the requirements of exercise intervention, but overall, the number of published articles is relatively small, which shows that there are relatively few studies on exercise and brain imaging in PD. In this exploratory research and data analysis based on brain imaging studies, the ALE guidebook-recommended threshold was set at *P* < 0.001, and the use of a non-corrected *P*-value method was used. As the number of neuroimaging studies in this field gradually increases, more literature can be included in the meta-analysis, and more stringent multiple comparison correction methods, such as false discovery rate (FDR) and family-wise error rate (FWE), can be verified to provide a more theoretical basis for exercise to promote the improvement of PD symptoms. The exercise intervention methods in this study involved various exercises, and the exercise duration and intensity are also inconsistent, but the results still show that exercise generally affects the brain activation patterns of PD. In conclusion, exercise for PD treatment enhances the activation of the cerebellum, occipital lobe, parietal lobe, and frontal lobe. The improvements conferred by exercise for PD do not cause a change in the activation of a single brain area but may result from coordinated changes in multiple brain areas.

## Data Availability Statement

The original contributions presented in the study are included in the article/supplementary material, further inquiries can be directed to the corresponding author/s.

## Author Contributions

RL, XF, and JL designed the study. JM, YW, LZ, JGu, WS, JGa, KS, ZL, and JZ collected the data. RL and JL analyzed the data and prepared the manuscript. All authors contributed to the article and approved the submitted version.

## Conflict of Interest

The authors declare that the research was conducted in the absence of any commercial or financial relationships that could be construed as a potential conflict of interest.

## Publisher’s Note

All claims expressed in this article are solely those of the authors and do not necessarily represent those of their affiliated organizations, or those of the publisher, the editors and the reviewers. Any product that may be evaluated in this article, or claim that may be made by its manufacturer, is not guaranteed or endorsed by the publisher.
